# Psycho-affective health, cognition, and neurophysiological functioning following sports-related concussion in symptomatic and asymptomatic athletes, and control athletes

**DOI:** 10.1038/s41598-021-93218-4

**Published:** 2021-07-05

**Authors:** V. Sicard, A. T. Harrison, R. D. Moore

**Affiliations:** 1 The Mind Research Network/Lovelace Biomedical Research Center, 1101 Yale Ave, Albuquerque, NM 87106 USA; 2 Arnold School of Public Health, University of South Carolina, 91 Assembly St, Columbia, SC 29208 USA

**Keywords:** Cognitive neuroscience, Biomarkers

## Abstract

Little is known about the neuropsychiatric and neurophysiological differences that characterize abnormal recovery following a concussion. The present study aimed to investigate the psycho-affective, cognitive, and neurophysiological profiles of symptomatic, slow-to-recover, concussed athletes, asymptomatic concussed athletes, and control athletes. Seventy-eight athletes (26 symptomatic, 26 asymptomatic, 26 control) completed the Beck Depression Inventory-II, Profile of Mood States, and 2-Back task. Additionally, event-related brain potentials were recorded during an experimental three-stimulus visual Oddball paradigm. Compared to asymptomatic and control groups, the symptomatic group reported greater depression symptoms and negatively altered mood states. Symptomatic athletes also exhibited poorer cognitive performance on the 2-Back task, indicated by more errors and slower reaction time. ERP analyses indicated prolonged P3b latency for both symptomatic and asymptomatic groups, but symptomatic athletes also exhibited reduced P3b amplitude compared to both asymptomatic and control groups. For the asymptomatic group, correlations were observed between time since last concussion and functioning, but no relations were observed within the symptomatic group for any measure. The current findings provide valuable information regarding the psycho-affective, cognitive, and neurophysiological profiles of athletes with and without persistent symptoms following a concussion and highlight the need to assess and treat symptomatic, slow-to-recover athletes from a multidimensional and integrative perspective.

## Introduction

Public awareness and media coverage of concussions have significantly increased in the past few years, largely due to heightened awareness of chronic conditions stemming from these brain injuries^1^. Concussive injuries involve a near-instant transfer of kinetic energy caused by either an impact or impulsive loading^2^. The insulting energy and shearing forces imparted to axons and the cerebral vasculature cause a complex sequence of pathophysiological processes, inducing acute cognitive dysfunction^3,4^. Although concussion effects are often considered to be transient, approximately 20–30% of individuals will exhibit a constellation of persistent physical (e.g., headache, dizziness), cognitive (e.g., difficulty concentrating, fatigue), and emotional (e.g., depression, anxiety) symptoms that can negatively impact social, academic, and vocational functioning^5^. There is a growing body of knowledge on the cognitive sequelae of concussions, yet we are just beginning to understand the psycho-affective and neurophysiological profiles of these symptomatic, slow-to-recover athletes^6^. Thus, one of the greatest clinical challenges is to determine whether persistent symptoms are reflective of concussion pathophysiology or socio-emotional factors, such as the emotional response to the injury.

Previous research indicates that athletes with a history of concussion who exhibit increased depression, anxiety, and other psycho-affective symptoms also exhibit alterations in neural structure, function, and connectivity^7–9^. These results suggest that the long-term sequelae of concussions are partly neurophysiological in origin. However, no study examined the psycho-affective, cognitive, and neurophysiological health of slow-to-recover athletes relative to both their injured but asymptomatic concussed peers and matched controls that never sustained a concussion. This is a major shortcoming, as understanding how these three groups differ may provide valuable insights regarding their clinical profiles and effective markers for injury prognosis and recovery.

Accordingly, the current study aimed to examine the psycho-affective, cognitive, and neurophysiological profiles of symptomatic, slow-to-recover athletes compared to athletes who sustained a concussion but were asymptomatic at the time of testing, and non-concussed controls. To achieve our aims, we employed the Beck Depression Inventory-II (BDI-II) and the Profile of Mood States (POMS) to measure psycho-affective health, a 2-Back task, and a three-stimulus visual Oddball Task to measure cognition, and we evaluated event-related potentials (ERPs) during the Oddball task to measure neurophysiological functioning. We predicted a stepwise pattern of results, whereby both symptomatic and asymptomatic groups would exhibit alterations in psycho-affective, cognitive, and neurophysiological health relative to the control group, but that the symptomatic group would exhibit the greatest alterations.

## Results

### Demographics

Demographic data are presented in Table 1. No group differences were observed for age, height, weight, body mass index, and years of education (*F*s﻿ ≤ 1.15, *ps* ≥ 0.64). The number of prior concussions or time since last injury did not differ between the symptomatic and asymptomatic groups, *t*s(76) ≤ 0.57, *ps* ≥ 0.50, suggesting that sample matching was successful.

**Table 1:** Summary demographic and symptom profiles for and symptomatic and asymptomatic concussed athletes, and non-concussed controls.

	Symptomatic (n = 26)	Asymptomatic (n = 26)	Control (n = 26)
Age	21.1 ± 2.8	21.0 ± 1.6	20.5 ± 1.5
Sex [M/F]	7/19	7/19	7/19
Handedness [RH/LH]	24/2	24/2	24/2
BMI	25.2 ± 4.9	27.0 ± 8.6	24.7 ± 8.0
Concussions (#)	1.9 ± 1.3 (range 1–4)	2.1 ± 1.4 (range 1–4)	–
Time Since Injury (days)	48.3 ± 19.4	46.4 ± 20.1	–
Education (years)	14.7 ± 2.5	14.8 ± 1.4	14.6 ± 1.4
Sports Played [F/S/R]	14/8/4	14/8/4	14/8/4
Total Symptom Score	27.7 ± 14.3	–	–
Mental	9.5 ± 4.5	–	–
Physical	8.5 ± 5.2	–	–
Emotional	5.2 ± 4.7	–	–
Sleep	4.4 ± 3.6	–	–

Means are reported with standard deviations.

Abbreviations: Sex [M, Males; F, Females]; Handedness [RH, Right-Handed; LH, Left-Handed]; Sport Played [F, Football; S, Soccer; R, Rugby].

### Psycho-affective measures

**Figure 1:**
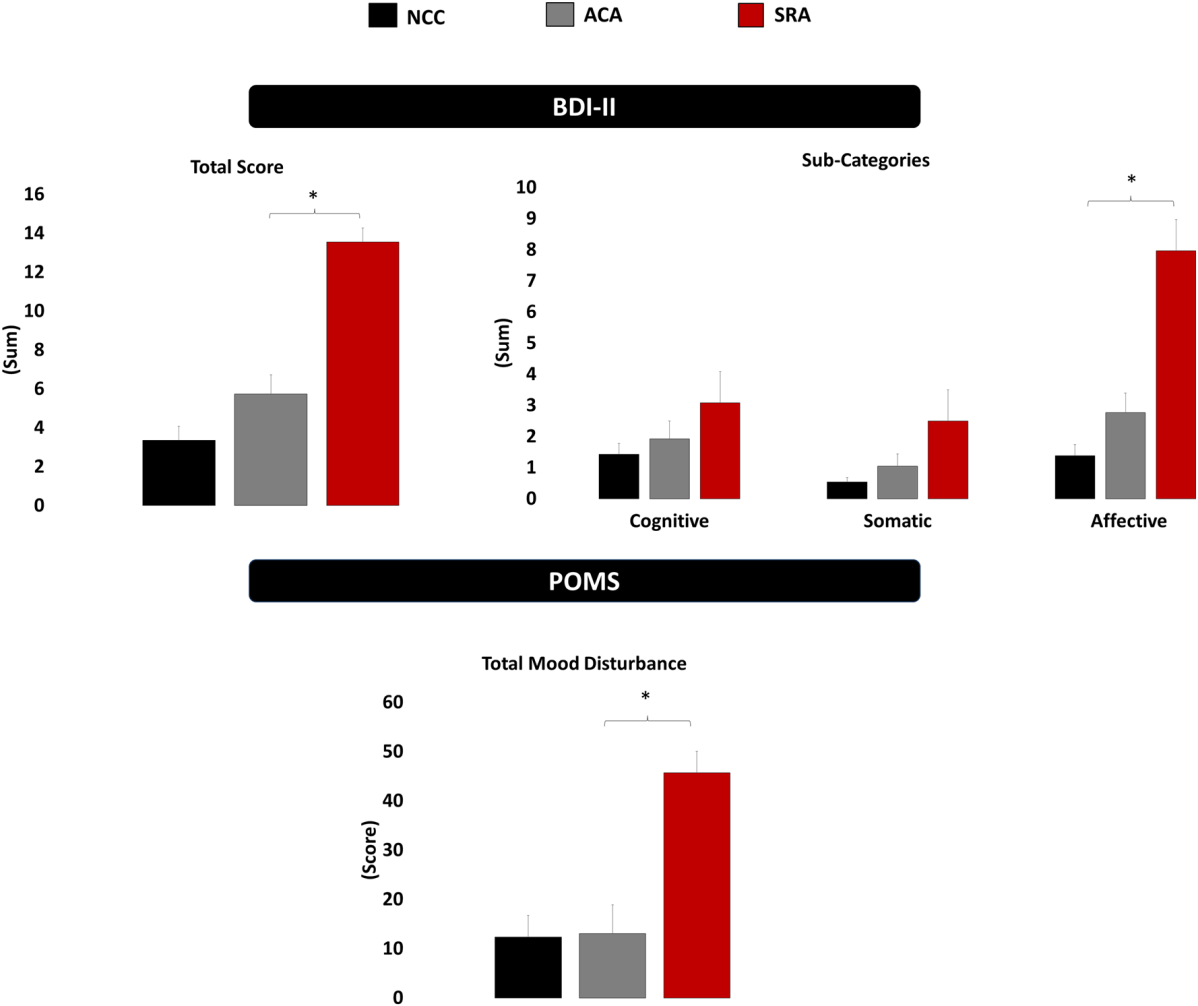
Psycho-affective outcomes measured by the Beck Depression Inventory-II (BDI-II) and Profile of Mood States (POMS) in the slow-to-recover athletes (SRA), asymptomatic concussed athletes (ACA), and non-concussed controls (NCC).

Data are presented as mean ± standard error. Significant psycho-affective findings are presented graphically in Fig. 1. The analysis revealed an effect of group for total depression score on the BDI-II, *F*(2,75) = 22.61, *p* < 0.01, with the symptomatic group (13.5 ± 1.3) reporting greater symptom score than the asymptomatic (5.7 ± 1.3) and control groups (3.5 ± 0.7; *ps* ≤ 0.01). Analyses of BDI-II sub-dimensions revealed group differences for both affective and somatic sub-dimensions, *F*s(2,75) ≥ 11.01, *ps* ≤ 0.01, with the symptomatic group reporting greater affective (symptomatic = 7.9 ± 0.6; asymptomatic = 2.7 ± 0.6; control = 1.4 ± 0.4) and somatic (symptomatic = 2.5 ± 0.4; asymptomatic = 1.0 ± 0.2; control 0.5 ± 0.1) symptoms of depression than both the asymptomatic and control groups, *ps* ≤ 0.01. No significant differences were observed between the asymptomatic and control groups for total depression scores or any depression sub-dimension (*ps* ≤ 0.01).

Analyses revealed an effect of group for total mood disturbance on the POMS, *F*(2,75) = 15.31, *ps* < 0.01, with the symptomatic group (45.7 ± 4.3) reporting greater mood disturbance than both the asymptomatic (13.1 ± 5.7) and control groups (12.4 ± 4.4; *ps* ≤ 0.01). Analyses of individual sub-scales revealed group differences for all mood states, *Fs*(2,75) ≥ 5.54, *ps* < 0.01, with the symptomatic group reporting greater tension/anxiety (symptomatic = 12.3 ± 1.5; asymptomatic = 7.3 ± 0.9; control = 8.0 ± 1.0), depression/dejection (symptomatic = 11.9 ± 1.4; asymptomatic = 6.0 ± 1.8; control = 4.3 ± 1.3), anger/hostility (symptomatic = 12.1 ± 1.7; asymptomatic = 5.8 ± 1.4; control = 3.9 ± 0.7), fatigue/inertia (symptomatic = 10.7 ± 1.0; asymptomatic = 5.3 ± 0.9; control = 6.3 ± 1.0), confusion/bewilderment (symptomatic = 10.0 ± 0.8; asymptomatic = 4.9 ± 0.9; control = 6.2 ± 0.7), as well as decreased vigor/activity (symptomatic = 11.2 ± 1.0; asymptomatic = 16.2 ± 1.0; control = 16.3 ± 0.8) than the asymptomatic and control groups (*ps* ≤ 0.03). No differences were observed between the asymptomatic and control groups (*ps* ≥ 0.54).

*S*ignificant relations were observed between time since injury, and both the total depression symptoms on the BDI-II and total mood disturbance on the POMS for the asymptomatic athletes (*rs* ≥ 0.48, *ps* ≤ 0.05). However, the same relations were not significant in the symptomatic athletes (*rs* ≤ 0.32, *ps* ≥ 0.11). The number of concussions was significantly related to depression/dejection symptoms on the POMS in both the symptomatic and asymptomatic groups (*rs* ≥ 0.51, *ps* ≤ 0.05). Within the symptomatic group, significant relations were observed between the number of concussions and total depression symptoms on the BDI-II and total mood disturbance on the POMS (*rs* ≥ 0.53, *ps* ≤ 0.05).

### Cognitive measures

**Figure 2:**
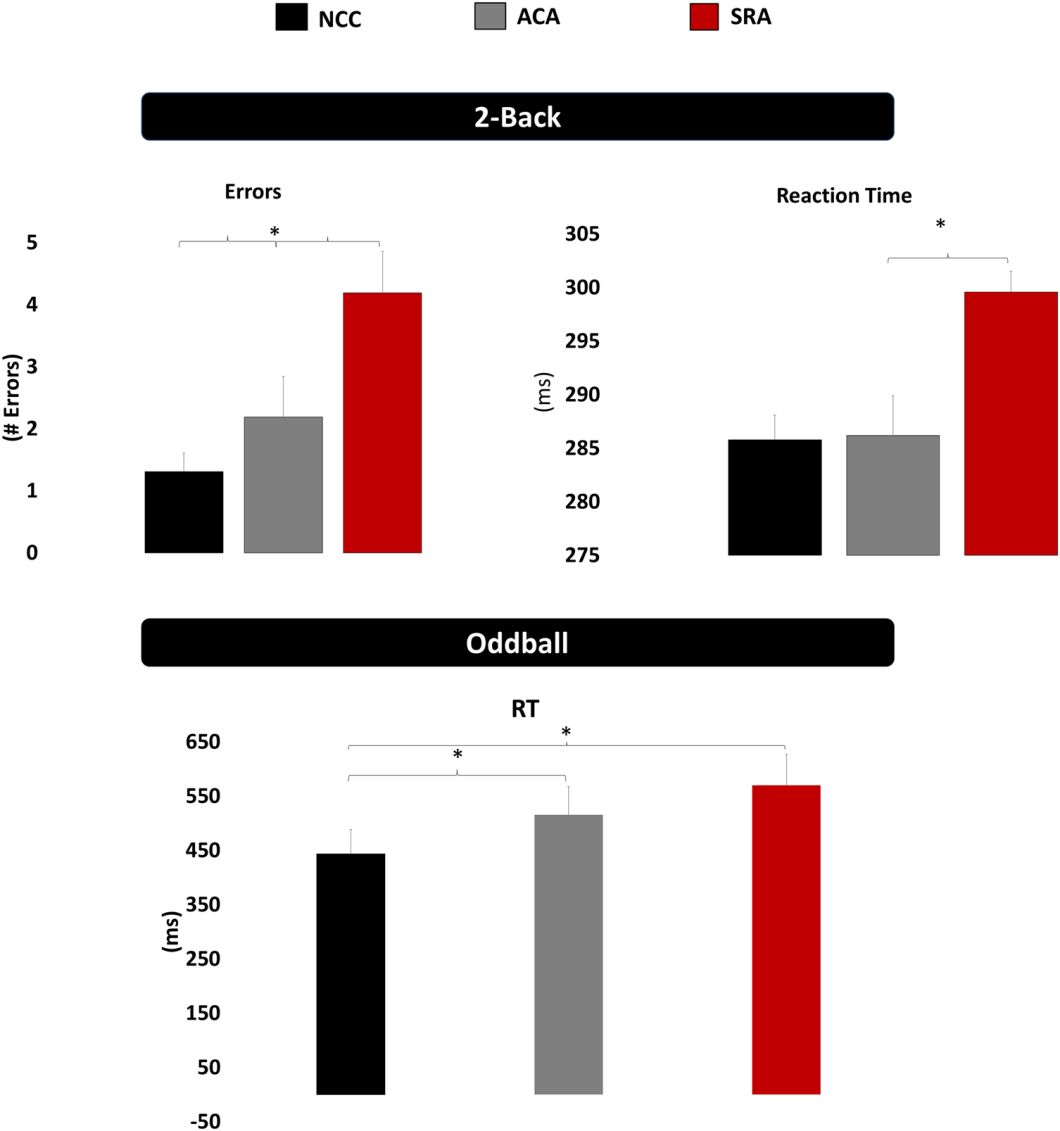
Cognitive outcomes on the 2-Back task (number of errors and reaction time) and the experimental visual three-stimulus Oddball task (reaction time) in the slow-to-recover athletes (SRA), asymptomatic concussed athletes (ACA), and non-concussed controls (NCC).

Data are presented as mean ± standard error. Significant cognitive findings are presented graphically in Fig. 2. Analyses of the 2-Back task revealed an effect of group for RT, *F*(2,75) = 8.01, *p* < 0.01, with the symptomatic group (3.0 ± 0.1) exhibiting slower RT than both the asymptomatic (2.86 ± 0.1) and control groups (2.87 ± 0.04; *ps* ≤ 0.01). No difference was observed between the asymptomatic and control groups (*p* = 0.99). Further, an effect of group was observed in response accuracy, *F*(2,75) = 6.89, *p* < 0.01, with the symptomatic group (4.2 ± 0.6) exhibiting decreased accuracy relative to both the asymptomatic (2.2 ± 0.7) and control groups (1.3 ± 0.3; *ps* ≤ 0.04). No difference was observed between the asymptomatic and control groups (*p* = 0.51).

Analyses of RT to the target stimulus on the Oddball Task revealed an effect of group, *F*(2,75) = 7.13, *p* < 0.01, with both the symptomatic (570.0 ± 13.1) and asymptomatic (515.9 ± 10.1) groups responding slower than the control group (444.1 ± 9.8; *ps* ≤ 0.01). However, the symptomatic group exhibited slower RT than the asymptomatic group (*p* = 0.04). Response accuracy failed to reveal any interaction or main effect, *Fs*(2,75) ≤ 1.85, *ps* ≥ 0.32.

*S*ignificant relations were observed between time since injury and accuracy and RT on the 2-Back Task, and RT on the Oddball task for the asymptomatic athletes (*rs* ≥ 0.62, *ps* ≤ 0.01). Again, the same relations were not significant in the symptomatic athletes (*rs* ≤ 0.26, *ps* ≥ 0.24). However, the number of concussions was associated with RT on the 2-Back Task within the symptomatic group (*r* = 0.55, *p* ≤ 0.05). Furthermore, for both the asymptomatic and symptomatic groups, the BDI-II cognitive symptoms correlated with RT on the 2-Back and Oddball tasks (*rs* ≥ 0.69, *ps* ≤ 0.01).

### EEG/ERPs

Preliminary analysis of ERP topography revealed variable sites of maximum activation around the midline in the central region (P3a) and parietal (P3b). In accordance with previous research^10–13^, 1, 106, 31, 80 (central); 61, 78, 67, 77 (parietal) regions of the international 10–5 system^14^ were created and subsequently analyzed. Data for three participants from each group were discarded due to excessive noise, leaving 23 participants for each group with ERP data. At least 35 trials were retained for ERP averaging for all athletes for the P3a and P3b. The number of trials retained for ERP averaging did not differ between groups for either the P3a or P3b, *Fs*(2,66) ≤ 1.51, *ps* ≥ 0.21.

**Figure 3:**
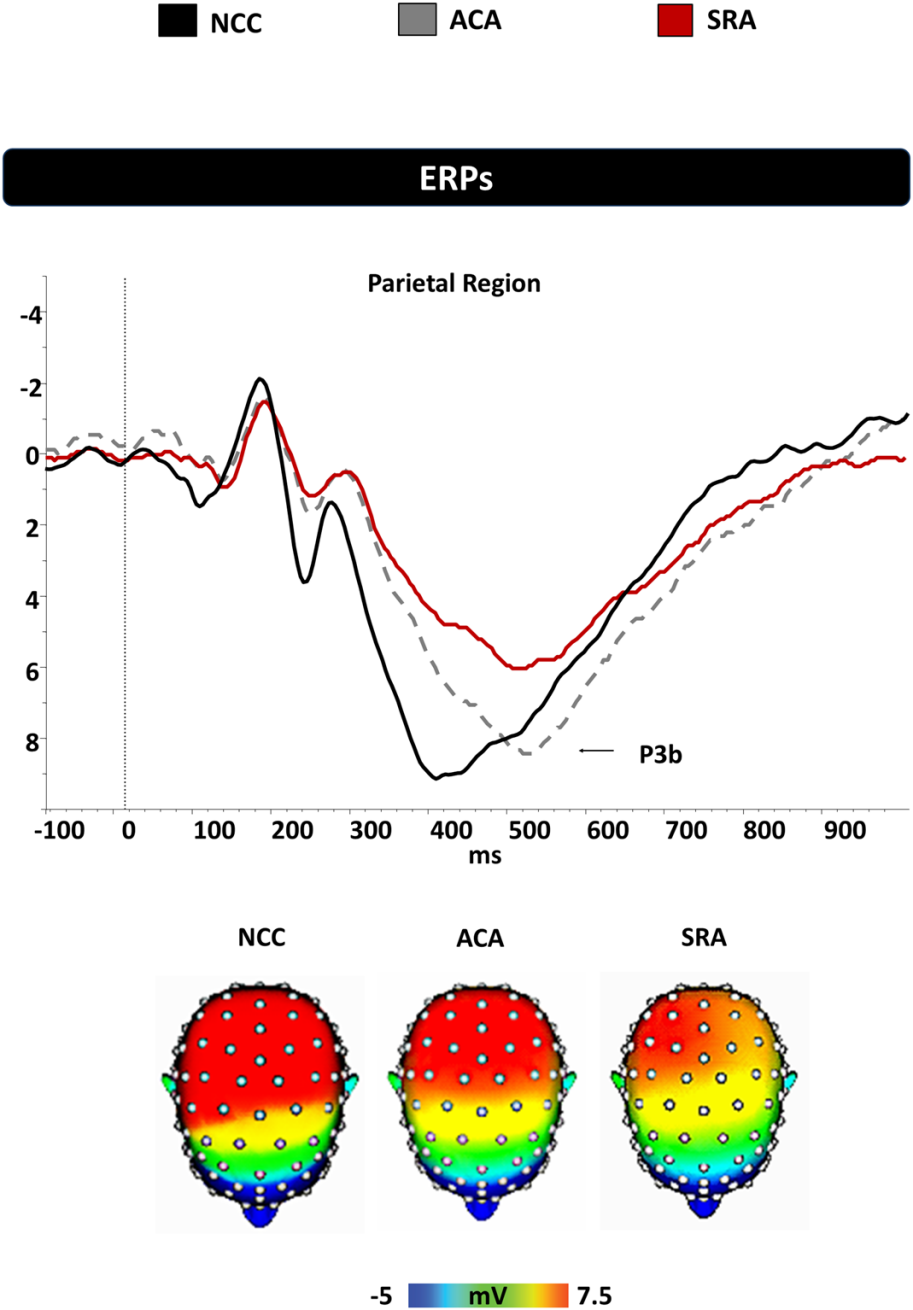
P3b component during the experimental visual three-stimulus Oddball task in the parietal region in the slow-to-recover athletes (SRA), asymptomatic concussed athletes (ACA), and non-concussed controls (NCC).

Data are presented as mean ± standard deviation. Significant ERPs waveforms and topographic maps are presented graphically in Fig. 3. Analyses evaluating the P3a failed to reveal an effect of group for amplitude or latency, all *Fs*(2,66) ≤ 1.10, *ps* ≥ 0.55. Analyses evaluating component latencies to target stimuli (P3b) revealed an effect of group, *F*(2,66) = 9.14, *p* < 0.01. Planned comparisons revealed that the symptomatic (508.4 ± 15.3) and asymptomatic (515.3 ± 13.3) groups exhibited longer P3b latencies than the control group (388.5 ± 16.7; *ps* < 0.01). The one-way ANOVA evaluating P3b component amplitude to target stimuli (P3b) revealed an effect of group, *F*(2,66) = 6.83, *p* = 0.02. Planned comparisons revealed that the symptomatic group (6.1 ± 2.1) exhibited decreased amplitude relative to the asymptomatic (8.2 ± 1.9) and control groups (9.1 ± 1.5; *ps* ≤ 0.03). No difference was observed between the asymptomatic and control groups (*p* = 0.31).

*S*ignificant relations were observed between time since injury and P3b latency in the asymptomatic athletes (*r* = 0.44, *p* < 0.05). Again, the same relation was not significant in the symptomatic athletes (*r* ≤ 0.16, *p* = 0.72). However, the number of injuries was significantly related to P3b amplitude in the symptomatic athletes (*r* = 0.67, *p* < 0.01). Within both the asymptomatic and symptomatic groups, significant negative relations were observed between BDI-II total score and cognitive sub-dimension and P3b amplitude (*rs* ≥ 0.69, *ps* < 0.01).

## Discussion

The current study sought to compare the psycho-affective, cognitive, and neurophysiological profiles among symptomatic, slow-to-recover athletes, athletes who sustained a concussion but were asymptomatic, and non-concussed controls. Symptomatic athletes reported worse emotional status, exhibited reduced cognitive performance, and showed decreased neurophysiological function relative to asymptomatic and control athletes.

Specifically, athletes in the symptomatic group reported greater depression symptoms than the asymptomatic or control groups. Importantly, our results suggest that the persistent depression symptoms measured in the symptomatic group cannot be solely attributed to somatic or cognitive sub-dimensions of depression, as differences were greatest for the affective sub-dimension. These depression symptoms may reflect an emotional reaction to the trauma and its sequelae, or some underlying pathological processes. Indeed, we also observed a strong correlation between depression symptoms and neurophysiological function in all athletes with a history of concussion. This is consistent with previous research indicating that athletes with depression symptoms exhibit decreased fMRI activation in prefrontal and striatum regions^15^, as well as significant alterations in frontal-EEG asymmetries^8^. Together with previous studies, our results suggest a physiological origin of the depression symptoms.

Athletes in the symptomatic group also reported greater total mood disturbance on the POMS than athletes in the asymptomatic and control groups, whereas no difference was observed between the symptomatic and control groups. These results are consistent with other studies showing athletes with persisting symptoms report greater mood disturbance than asymptomatic or non-injured athletes^16–18^. Moreover, correlational analyses revealed that the number of concussions was significantly related to depression/dejection symptoms in both the symptomatic and asymptomatic groups. Significant relations were also observed between time since injury and total mood disturbance for the asymptomatic group. However, the same relations were not significant in the symptomatic group, suggesting that the athletes from the symptomatic group have altered psycho-affective recovery profiles compared to asymptomatic athletes.

Together, the results on the BDI-II and POMS support the argument for going beyond standard concussion symptoms scales (i.e., SCAT), which only ask a few questions about psycho-affective health and only provide a global score. As noted by the consensus of the Concussion in Sport Group^1^, these scales are of little utility beyond 48–72 h. Unfortunately, most clinicians and researchers still rely on concussion symptom scales to gauge the psycho-affective recovery of individuals with a concussion. Further implementation of validated and easy-to-administer psychometric scales of mental health such as the BDI-II and POMS will help researchers and clinicians more accurately identify and track psycho-affective alterations in a comprehensive manner.

Athletes in the symptomatic group also exhibited multifaceted cognitive alterations relative to asymptomatic and control groups indexed by longer reaction times and decreased response accuracy during the 2-Back task. Both accuracy and RT were significantly related to time since injury for the asymptomatic group, but not the symptomatic group. These results are further suggestive of a different recovery profile for symptomatic athletes and indicate cognitive deficits are not remitting over time for symptomatic athletes. Furthermore, cognitive performance was related to cognitive symptoms of depression, but not affective or somatic symptoms, for both symptomatic and asymptomatic athletes. This indicates that cognitive performance was not influenced by general depressive symptoms in either concussion groups and cognitive differences between symptomatic and asymptomatic athletes cannot be attributed to differences in depressive symptoms. Our results add to a growing body of research observing working memory impairments in symptomatic, slow-to-recover athletes^19–27^. As working memory deficits are associated with multiple neurocognitive disorders, as well as decreased academic achievement and vocational attainment^28^, these deficits may have pervasive influences. This is particularly cogent in symptomatic athletes, as correlational analyses imply working memory deficits are not remediating as a function of time since last concussion.

Both the symptomatic and asymptomatic group exhibited prolonged response times during the Oddball task, but the symptomatic athletes exhibited the greatest delays. Previous research indicates that concussive injuries can lead to deficits in visual processing and sustained attention^12,13,29^, critical processes underlying successful performance in the Oddball task. Thus, even under relatively low cognitive loads, concussions may be associated with delays in visual processing speed weeks-to-months following injury. Additionally, correlational analyses revealed significant relations between time since injury and RT on the Oddball task for asymptomatic athletes, but not the symptomatic athletes, further highlighting the decoupling of normal recovery processes for the symptomatic group. In combination with the results from the 2-Back task, these results suggest that symptomatic athletes may need cognitive rehabilitation to regain efficient cognitive functioning.

Athletes in the symptomatic and asymptomatic groups exhibited longer P3b latencies during the Oddball task relative to the control group. The P3b latency is believed to reflect stimulus evaluation and classification speed independent of response selection^30^. Thus, irrespective of symptomology, all athletes with a concussion exhibited neural processing delays relative to controls, however, the symptomatic group exhibited the longest latencies. Again, correlational analyses revealed significant relations between time since injury and P3b latency for athletes in the asymptomatic group, but not in the symptomatic group. Thus, a consistent pattern is present across the psycho-affective, cognitive, and neurophysiological domains, whereby normal recovery with time is not present in athletes with persistent symptoms.

In addition to latency, athletes in the symptomatic group exhibited reduced P3b amplitudes relative to the asymptomatic and control groups. The number of concussions was inversely related to P3b amplitude for the symptomatic athletes, indicating an accumulative detriment of concussive injuries on neurophysiological health. The P3b component, evoked in response to the infrequently occurring target stimulus, is believed to reflect the revision of mental events, with amplitude being proportionate to the degree of attention resources allocated during stimulus engagement^30,31^. Prior research also observed that in comparison with control athletes, symptomatic athletes who sustained a concussion exhibit a reduction in P3b amplitude in the visual and auditory domains^32^. Thus, the current findings support prior observations that cognitive alterations in athletes with persistent symptoms appear to be of a neurophysiological origin^7,15,19,20,22,23,25–27,33,34^. However, correlations were observed for both the symptomatic and asymptomatic groups between BDI-II total symptom scores and cognitive symptoms sub-scores and P3b amplitude, highlighting the complex interaction between psycho-affective and cognitive health.

Although guidelines for field care and the graduated return to school and return to play are well-defined, the guidelines for athletes experiencing persistent symptoms are less clear. A greater understanding of what underlies abnormal recovery is necessary to improve the assessment, management, and rehabilitation of symptomatic, slow-to-recover athletes. This study suggests that slow recovery is not a one-dimensional concept, but a multidimensional cluster of psycho-affective, cognitive, and neurophysiological alterations. Accordingly, a multidimensional and integrative perspective is needed to assess, manage, and rehabilitate symptomatic athletes.

Currently, many clinicians rely on concussion questionnaires such as the Sport Concussion Assessment Tool-5 (SCAT-5) to assess individuals in a clinical setting. As the name implies, these questionnaires are meant for sideline and locker-room evaluations, and not full clinical evaluations. As suggested by the current findings, chronic assessment of sport-related concussions may benefit from easy-to-administer and validated psycho-affective assessments such as the BDI-II and POMS. Moreover, the use of computerized test batteries is highly recommended by the Consensus Statement on Concussion in Sport as well as the National Institute of Neurological Disorders and Stroke and Department of Defense^35,36^. Such batteries are easy to implement and provide results within minutes. Multiple test batteries are commercially available, such as ImPACT, DANA, CNS-VS, ANAM4, and Cogstate, yet studies have suggested that the Cogstate and CNS-VS are the most reliable and useful beyond the acute phase of injury^37–41^.

Furthermore, researchers and clinicians rarely implement actual measures of brain function to evaluate brain injury. This has been largely due to the lack of access, prohibitive cost, and the extensive expertise required for such measures. Fortunately, the cost of EEG machines is decreasing rapidly and available systems such as the eVox Brain Health System^42^ provide automated assessments of the P3a and P3b ERPs, as well as other useful EEG metrics. Importantly, these assessments (along with computerized cognitive assessments) are now reimbursable through many insurance plans making these systems feasible for clinical use.

Future researchers and clinicians should adopt multidimensional assessments implementing more comprehensive and validated measures of psycho-affective health, and, whenever possible, measures of neurophysiological functioning. Doing so will not only advance the scientific understanding of concussion recovery but also advance clinical assessment and management of concussive injuries.

Although the current study makes advances on prior research and is characterized by several strengths, the results must be interpreted considering the following limitations. First, the cross-sectional nature of this study prevents causative conclusions, and it is possible that some unmeasured variables contributed to the current group differences. However, we tried to minimize this possibility by carefully matching groups for age, years of education, sport, and injury characteristics, and we excluded participants who had a history of neurodevelopmental, neurological, or neuropsychiatric disorders. Further, premorbid intellectual abilities were not assessed in the present cohort, which may have influenced cognitive recovery. The current protocol also prevents the understanding of how psycho-affective symptoms manifested within each group across multiple phases of injury. Although all participants were free of a history of mood disorders, we did not have access to pre-injury and acute injury data, and the long-term removal from play can negatively influence athletes’ mood states. Finally, classification into the symptomatic and asymptomatic groups is based on self-reported symptoms and the under-reporting of symptoms is always a concern^43^. However, the distinct psycho-affective, cognitive, and neurophysiological profiles of athletes in the symptomatic and asymptomatic groups indicate that the groups objectively differed across multiple domains even when employing the most conservative correction procedure for post hoc tests to minimize the possibility of type I error.

Irrespective of limitations, the current study provides valuable information regarding the psycho-affective, cognitive, and neurophysiological profiles of athletes with and without persisting post-concussion symptoms and highlights the need to assess and treat slow-to-recover athletes from a multidimensional and integrative perspective.

## Methods

### Participants

Seventy-eight athletes (26 symptomatic concussed athletes; 26 asymptomatic concussed athletes; 26 non-concussed controls) were recruited from university football, rugby, and soccer teams to participate in this study. Data were collected in concert with a sports medicine clinic per the Declaration of Helsinki and data analyses were approved by the University of South Carolina Institutional Review Board. Prior to testing, all participants were made aware of the purpose and procedures, and informed consent was obtained. For their known relation with cognitive outcomes of concussion, exclusion criteria for all participants included substance abuse, special education, having a prior diagnosis of psychiatric or neurological disorders, or learning disabilities^44–46^. All participants had normal or corrected-to-normal vision, and no athlete had undergone the psycho-affective, cognitive, or neurophysiological assessment prior to the study.

To control for variability in injury diagnosis and age at injury for both concussion groups, only athletes incurring their concussion(s) during university sports were included in this study. All concussions were identified on-field by the team medical staff and diagnosed within 24 h of injury by the team physician using the criteria established by the American Academy of Neurology^47^ and Concussion in Sports Group^48^.

The symptomatic group included athletes who incurred a concussion but had not returned to play due to persistent symptoms. In contrast, athletes in the asymptomatic group made a complete return to play within three weeks of injury after completing the progressive Zurich Guidelines^1^. The control group included teammates who never incurred a sport- or non-sport-related brain injury. These athletes were carefully screened to ensure that they had not experienced an undocumented concussion, and they were automatically excluded from the study if they reported ever experiencing any of the symptoms listed on the Sport Concussion Assessment Tool-3 (SCAT-3) following a blow to the body, neck, or head. To minimize the influence of a priori variables, participants from the three groups were matched for age, years of education, BMI, sports played, and years of sports participation.

### Procedures

Participants first completed a semi-structured questionnaire to confirm their history of concussions and to exclude controls who may have sustained an undiagnosed concussion. Following the semi-structured interview, participants completed the Beck Depression Inventory-II (BDI-II)^49^, and the Profile of Mood States (POMS)^50^. Participants were then guided into an electrically shielded testing chamber where they completed the 2-Back task from a modified Cogstate battery^51^. Participants were then fitted with a 128-sensor high-density electroencephalographic (EEG) net and they completed a three-stimulus (standard, non-target, target) visual Oddball task while event-related brain potentials (ERPs) were recorded. Participants were given brief breaks between each test and total testing time was approximately 2 h.

### Psycho-affective measures

The Beck Depression Inventory-II (BDI-II). The BDI-II^52^ consists of 21 questions designed to assess the presence and intensity of cognitive, affective, and somatic symptoms of depression^53^. For each question, participants were asked to choose between four statements (scored 0 to 3), which best described how they have felt over the past two weeks. Scores range from 0–21 for cognitive, 0–15 for affective, and 0–27 for somatic sub-dimensions. A total score, ranging from 0 to 63, was obtained by adding the answers to each question, with higher score indicating greater intensity of depression symptoms^54^. The BDI-II demonstrates high reliability, validity, and sensitivity in non-clinical and clinical populations, including those with concussion^49,55^.

The Profile of Mood States (POMS). The POMS^50^ is a 65-item checklist designed to assess tension/anxiety, depression/dejection, anger/hostility, fatigue/inertia, vigor/activity, confusion/bewilderment, as well as total mood disturbance. Participants used a 5-point Likert scale starting from 0 (not at all) to 4 (extremely) to report how they have felt over the past week. Scores range from 0–36 for tension/anxiety, 0–60 for depression/dejection, 0–48 for anger/hostility, 0–28 for fatigue/inertia, 0–32 for vigor/activity, and 0–28 for confusion/bewilderment. Scores from individual subscales were used to calculate the total mood disturbance by subtracting positive mood score (i.e., vigor/activity) from the sum of negative mood scores. The POMS has shown high reliability and validity and is used to characterize overall and categorical mood disturbances in non-clinical and clinical populations, including those with a concussion^8,56^.

### Cognitive measures

2-Back Task. Participants completed the 2-Back task from the Cogstate test battery due to the known relation of concussive history to executive functions^12,57–61^. A playing card is presented face up in the center of the screen. If the card presented is identical to the one presented two cards previously, the participant was instructed to press the “yes” key; if it is not the participant was instructed to press the “no” key. Outcome measures were accuracy (i.e., percentage of good responses) and average reaction time on accurate trials.

Oddball Task*.* Participants completed a three-stimulus visual Oddball task, a task designed to assess levels of sustained attention. This Oddball task is sensitive to the persistent effects of concussion^10,62^. The task consisted of a 3.5 cm diameter “standard” circle, a 5 cm diameter “target” circle, and a 5 cm wide “non-target” square, all of which appeared on a black background. The standard stimulus occurred on 76% of trials, the target occurred on 12% of trials, and the non-target occurred on 12% of trials. All stimuli were presented focally for 70 ms and the inter-stimulus interval ranged from 1600 to 2000 ms. Participants sat one meter away from the computer screen in a faradized room. Participants were instructed to respond as quickly and accurately as possible with the index finger of their dominant hand only when the target stimulus appeared. Participants completed 30 practice trials followed by three test blocks of 160 trials each. Rest periods of two minutes were provided between each block. Total task time including rest periods was approximately 20 min.

### EEG/ERPs

Continuous EEG was recorded with a 128-sensor Geodesic EEG Sensor Net (EGI, Eugene, OR, USA). Site impedance was kept below 50 kΩ, which is an acceptable level for high input impedance amplifiers^63^. Each site was referenced online to site Cz. Additional sensors were placed above, below the left orbit, and on the lateral canthus of each eye recorded electro-oculographic (EOG) activity. The EEG signal was amplified with Net Amps 200 amplifier (EGI, Eugene, OR, USA) and a band-pass filter was set at 0.1–100 Hz. The signal was digitalized at 250 Hz and the data were recorded with Net Station software (EGI, Eugene, OR, USA).

Offline data reduction was performed with Brain Vision Analyzer version 2.10 (Brain Products GmbH, Munich, Germany). Eye movement artifacts were corrected with the Gratton and Coles algorithm^64^, and data were re-referenced to the whole head average. Atypical EEG artifacts were semi-automatically inspected and segments containing EEG activity exceeding ± 100 µV were rejected. Before averaging, trials for which participants gave an incorrect response, or with a reaction time exceeding ± 2.5 SD, were rejected.

Isolation of ERP components included the creation of epochs from 100 ms pre-stimulus baseline and to 1000 ms after stimulus onset. Epochs were filtered with a 30 Hz (24 dB/octave) high-pass filter. The P3a component was obtained by identifying the mean amplitude within a 50 ms time window surrounding the largest positive-going peak within the interval 250–450 ms after stimulus onset at fronto-central sites. Similarly, the P3b component was identified as the mean amplitude within a 50 ms interval surrounding the largest positive-going peak within the interval 300–700 ms after stimulus onset at centro-parietal sites.

### Statistical analyses

Demographic data, total scores and sub-scales for the BDI-II and POMS, and the clinical variables of the 2-Back task (response time [RT] and accuracy) were analyzed by a series of one-way ANOVAs. For the Oddball task, response accuracy was evaluated by a 3 × (Group: symptomatic, asymptomatic, control) × 2 (Target: target, non-target) repeated measures ANOVA. Response time to target stimuli was evaluated by a one-way ANOVA (Group: symptomatic, asymptomatic, control). Amplitude and latency for P3a and P3b were analyzed by a series of one-way ANOVAs.

Bivariate correlations were conducted to evaluate the relations between time since injury, number of prior concussions, and significant indices of psycho-affective, cognitive, and neurophysiological function. All within-subject effects are reported according to Greenhouse–Geisser’s correction. Significant interactions were evaluated by independent t-tests or pairwise comparisons, as appropriate, using the Bonferroni correction. An *α* = 0.05 was used for all tests. All statistical analyses were completed with SPSS 22.0 for Windows (IBM, Chicago, United States).

## Data Availability

The datasets generated and analyzed during the current study are not publicly available due to IRB regulations but are available from the corresponding author on reasonable request.
